# Development and Evaluation of Preharvest *Thaumatotibia leucotreta* Citrus Fruit Infestation Monitoring for Inclusion in a Systems Approach

**DOI:** 10.3390/insects16060589

**Published:** 2025-06-03

**Authors:** Sean D. Moore, Tamryn Marsberg, Mellissa Peyper, Luke Cousins, Marcel van der Merwe, Guy Sutton, Sonnica van Niekerk, Vaughan Hattingh

**Affiliations:** 1 Citrus Research International, 91 Heugh Road, Walmer, Gqeberha 6070, South Africa; tammy@cri.co.za (T.M.); mpeyper@cri.co.za (M.P.); luke@cri.co.za (L.C.); sonnica@cri.co.za (S.v.N.); 2 Centre for Biological Control, Department of Zoology and Entomology, Rhodes University, Life Science Building, Barrat Complex, African Street, Makhanda 6139, South Africa; g.sutton@ru.ac.za; 3 Department of Biochemistry and Microbiology, Rhodes University, Biological Sciences Building, Artillery Road, Makhanda 6139, South Africa; m.vandermerwe@ru.ac.za; 4 Citrus Research International, Department of Horticultural Science, Stellenbosch University, Stellenbosch 7600, South Africa; vh@cri.co.za

**Keywords:** false codling moth, citrus pest management, preharvest monitoring, sanitation sampling, systems approach, phytosanitary inspection, infestation assessment

## Abstract

*Thaumatotibia leucotreta*, an important citrus pest in southern Africa, is subject to phytosanitary regulations for certain export markets. A systems approach has been developed as an alternative to standalone postharvest disinfestation methods, integrating multiple risk mitigation steps, including preharvest infestation monitoring. This study aimed to validate an existing preharvest monitoring protocol based on fallen fruit collected under designated data trees and to develop a novel monitoring system based on sampling of sanitation fruit. Monitoring was conducted in seven Navel orange orchards (N = 7 each year) during the 2021 and 2022 seasons, representing high and low infestation levels, respectively. Infestation levels were assessed over 11–12 weeks by inspecting fruit beneath four sets of five trees and all sanitation fruit collected per orchard. The new system, which involves inspecting a 100-fruit sample per orchard, was compared with the previous method. While the five-tree protocol tended to overestimate infestation, it remained effective. The sanitation-fruit sampling approach accurately reflected orchard-level infestation, with a 100-fruit sample sufficient for orchards as large as 20 hectares. Although random sampling is recommended, it was not essential for effectiveness. These results support the use of the new monitoring procedure in the systems approach for citrus exports for *T. leucotreta* risk mitigation.

## Simple Summary

The false codling moth is an important pest that can attack citrus fruit in southern Africa. Because this pest can infest fruit, it poses a threat to citrus exports, especially to countries with strict import rules. To reduce the risk of exporting infested fruit, farmers follow a “systems approach”, which includes several steps to keep pest levels low. One of these steps is checking for signs of the pest before harvest. This study looked at two ways of achieving this: an older method that involves checking fruit that have fallen from a few specific trees, and a new method that involves inspecting fruit collected during orchard clean-up. This research was carried out over two seasons, one with high pest levels and one with low levels. Both methods proved to be effective and acceptably accurate. The new method, which uses a sample of 100 fruit per orchard, was more reliable and worked well for orchards up to 20 hectares in size. These results support the use of the new method as part of the systems approach, helping growers reduce the risk of pest damage and maintain access to valuable international markets.

## 1. Introduction

South Africa is the second largest exporter of citrus globally, and the largest global shipper of fresh citrus fruit. South Africa accounts for around 10% of global citrus fresh fruit exports, with approximately 71% of South Africa’s citrus production being exported [1,2]. Europe is the largest market for South African citrus exports, comprising 36% of the country’s export volumes [1]. *Thaumatotibia leucotreta* (Meyrick, 1913) (Lepidoptera: Tortricidae), also known as false codling moth (FCM), is endemic to sub-Saharan Africa [3] and is a regulated phytosanitary pest for certain export markets, including the European Union (EU) since January 2018 [4]. Effective *T. leucotreta* infestation risk mitigation procedures are accordingly required to sustain exports. A systems approach for *T. leucotreta* [5,6] was developed and implemented as an alternative to standalone postharvest disinfestation treatments. The use of systems approaches is guided by the International Standards for Phytosanitary Measures (ISPM 14) of the International Plant Protection Convention (IPPC) [7], as a procedure/treatment to manage the risk of quarantine pests, and its use is provided for in EU phytosanitary regulations [4,8,9]. A systems approach is defined as the integration of control measures, which may entail preharvest and postharvest actions, with at least two measures working independently from one another to provide adequate quarantine security [7,10]. The development of a systems approach for *T. leucotreta* was reported by Moore et al. [5], using three independent measures, including 13 distinct components or steps. This system was officially implemented by South Africa in 2018 for the export of fresh citrus from South Africa to the EU. The system was later validated and improved by Hattingh et al. [6], who demonstrated the system to be at least as effective as a standalone Probit 9 efficacy disinfestation treatment. The three measures in the systems are as follows: (1) preharvest controls and measurements, post-picking sampling, inspection and packing house measures; (2) post-packing sampling and inspection; and (3) shipping conditions [6].

One of the components within Measure 1 of the systems approach that can be used to categorise the phytosanitary status of the orchard is the monitoring of fruit infestation in the orchard for at least the last 12 weeks before harvesting begins, with associated infestation thresholds [5,6]. If the threshold is exceeded during the period 12 to 4 weeks before start of harvest, the orchard requires a corrective treatment, and if it is exceeded during the last 4 weeks before harvesting begins, the range of available shipping condition options is reduced to provide a higher level of risk mitigation. In the monitoring system originally used in the systems approach, fallen fruit from underneath five data trees per orchard were collected and analysed during the 12 week period before start of harvesting [6], with a sliding scale for additional sets of five data trees per orchard applied to increasing orchard size (unpublished systems approach protocol). It was recommended that the data trees be placed together and positioned in the orchard wherever fruit drop shows the highest level of infestation. The infestation threshold was originally set at an average of more than 0.2 infested fruit per tree per week [5,6], but was subsequently revised to an average of no more than 0.1 infested fruit per tree per week (unpublished systems approach protocol).

Although the *T. leucotreta* infestation level recorded preharvest is not itself used in calculating the efficacy of the systems approach [6], the threshold associated with the monitoring system contributes to the categorisation of the phytosanitary status of the orchard, which in turn influences the range of options available in subsequent Measures 1 and 3, and these are efficacy calculation parameters. It is therefore important that the sensitivity and accuracy of the preharvest infestation monitoring system be validated and potential for improvements evaluated. The need for improvement has been indicated by concern that the original system is vulnerable to inadvertent interference when orchard sanitation practices do not effectively exclude the data trees, resulting in unintended removal of fallen fruit from underneath the data tress.

Consequently, this study was conducted to evaluate the accuracy (sensitivity and representivity) of the current in-orchard preharvest infestation monitoring method and to evaluate potential improvements to the monitoring system. The appropriateness and usefulness of the measurement of pest level, through sampling, were determined according to their ability to statistically approximate (estimate) the pest population in the orchard as a whole.

## 2. Materials and Methods

### 2.1. Trial Sites

The study was conducted in the Sundays River Valley in the Eastern Cape Province of South Africa over two successive seasons. In the first season (2021), 10 Navel orange orchards ranging from 1.30 to 1.48 ha in size, with a history of notable *T. leucotreta* infestation were selected (Table 1). This number was later reduced to seven orchards, as it became clear that even with intensive labour, this was the maximum number of orchards manageable for the study. In the second season (2022), the study was conducted in seven different orchards. The orchards selected ranged from 1.02 ha to 2.58 ha. Tree spacing in all orchards used over the two seasons was 6 m (trees) × 3 m (rows), which is the most common planting spacing used in the region. Contrary to the first season, in this second season, orchards with a historically low *T. leucotreta* infestation were selected. This was purposefully implemented to include both high and low *T. leucotreta* presence orchards in evaluating the monitoring systems.

### 2.2. Trial Layout and Evaluation

**Figure 1:**
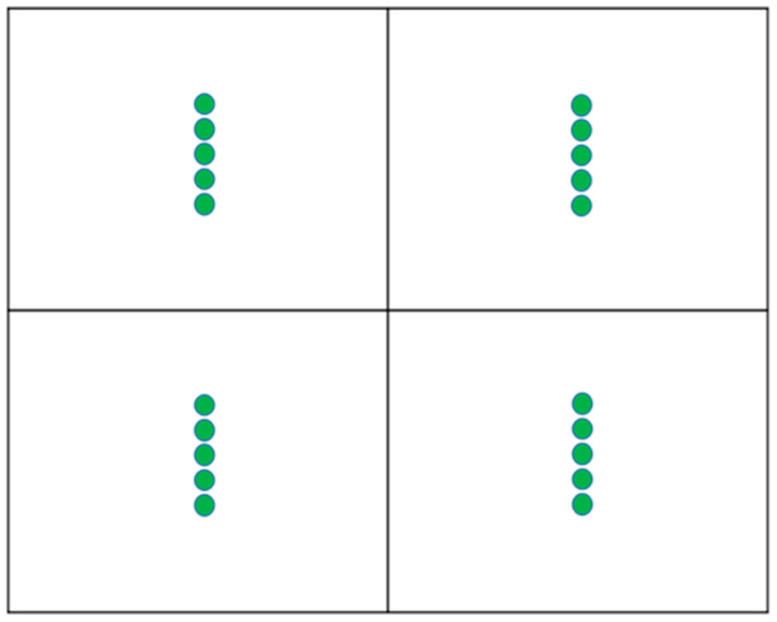
Layout of the trial within an orchard, with one set of five data trees (the green dots) in each of four quadrants in the orchard.

Each orchard was divided into four quadrants (Figure 1). Five data trees were selected, marked and cordoned off with hazard tape in each of the quadrants for each orchard. This was carried out not only for the easy identification of data trees but also to ensure that farm sanitation teams did not collect fruit under these data trees. Quadrant division was easy in the first season, as orchards were relatively square. However, in the second season, orchards were irregular in shape, making the sectioning of orchards more variable. From a projected 12 weeks before harvest (15 April 2021 and 11 April 2022), fallen fruit were collected from each of these data trees and analysed for *T. leucotreta* infestation. This was carried out twice a week. All *T. leucotreta* infested fruit were abscised by the tree and drop to the ground [11]. The collection of fallen fruit was therefore considered an absolute and accurate data collection method, not being subject to sampling error or bias [12]. Infestation was recorded by dissecting fruit with a sharp knife and determining the presence of *T. leucotreta* larvae, or signs of tunnelling and frass within the fruit, indicative of the larva having exited [11]. On the same days, sanitation teams would collect all fallen fruit from the entire orchard. These fruit were also assessed for *T. leucotreta* infestation in the same way. This continued twice weekly, until shortly before harvest (late June in 2021 and mid-July in 2022). To determine *T. leucotreta* infestation per tree per week, infested fruit that had fallen since the fruit collection 7 days prior, were divided by the total number of trees in the orchard.

### 2.3. Experimental Objectives and Associated Statistical Analysis

Four questions were addressed and statistically analysed, using the data generated from this trial, with the objective of determining the accuracy of the five-data tree preharvest infestation monitoring protocol within the *T. leucotreta* systems approach and to develop a potentially more reliable monitoring method.

#### 2.3.1. *Thaumatotibia leucotreta* Infestation from Data Trees as a Measurement of Infestation in the Orchard as a Whole

Note that the value of the pest population level measurement, here and throughout the manuscript, is determined according to its ability to statistically approximate (estimate) the population in the whole orchard. A generalised linear mixed modelling (GLMM) approach was used to evaluate whether *T. leucotreta* infestation rates in data-trees were correlated with infestation rates in sanitation fruit from the whole orchard. *Thaumatotibia leucotreta* infestation levels determined through the inspection of all the sanitation fruit from the whole orchard were modelled as a function of the *T. leucotreta* infestation levels derived from the data trees and the time since the start of the growing season (in weeks). A multiplicative term between *T. leucotreta* infestation from the whole orchard’s sanitation fruit and week was included in the model formulation to allow the magnitude of the correlation with *T. leucotreta* infestation levels derived from the data trees to vary over the growing season. A nested random intercept term of orchard/tree was included to account for repeated *T. leucotreta* infestation measurements derived from the same orchard and tree over the growing season [13]. The model was specified assuming a Gaussian error distribution and an identity link function. Hypothesis testing was performed using a Wald’s test (*p* < 0.05). The strength of the correlation between *T. leucotreta* infestation levels derived from the data trees and *T. leucotreta* infestation from the sanitation fruit from the whole orchard over time was quantified using the conditional R2 value from the ‘partR2′ R package [14].

A similar approach was adopted to assess whether the *T. leucotreta* infestation levels derived from data trees exceeding a threshold of 0.1 infested fruit/tree/week was predictive of *T. leucotreta* infestation in the sanitation fruit from the whole orchard also exceeding 0.1 infested fruit per tree per week. To do so, both the data tree and whole orchard measurements were converted into binary variables, where if the estimated *T. leucotreta* infestation level was above the threshold of 0.1 infested fruit/tree/week, it was scored as a 1, and if the estimate was below the threshold, it was scored as a 0. Thereafter, the threshold *T. leucotreta* measurements from the data-tree monitoring were modelled as a function of the threshold *T. leucotreta* measurements from the whole orchard sanitation fruit and the time since the start of the growing season (in weeks). The model was specified assuming a binomial error distribution and a logit link function. The marginal probability of the *T. leucotreta* infestation rate in the whole orchard (sanitation fruit) being above threshold, given that the *T. leucotreta* infestation rate in the data tree sample was above threshold, was calculated using the ‘ggeffects’ R package (ver. 2.2.1) [15].

#### 2.3.2. Sample Size Determination from Sanitation Fruit for Measuring *T. leucotreta* Infestation in the Total Sanitation Fruit Population from an Orchard

To assess the size of the fruit samples required to measure the *T. leucotreta* infestation levels per orchard based on the full sanitation population, a Monte Carlo resampling simulation was performed. Appropriate sample sizes were calculated based on accuracy, by assessing the random sample sizes required that the sub-sample measure approximated as the true population parameter (following van Steenderen et al. [16]). Here, the true population parameter was defined as the measured *T. leucotreta* infestation level per orchard/week based on the full sanitation sample. A simulation array was constructed that defined the random fruit samples, with replacement, per orchard/week, and repeated this process for 9999 iterations. Sampling adequacy was calculated as whether the fruit sample measurement was above the lower bound of the 95% confidence interval of the true *T. leucotreta* density measurement per orchard/week or not. Thereafter, sampling coverage was defined as one minus the proportion of iterations per sample size per orchard per week, where the sub-sample *T. leucotreta* infestation level measurement approximated *T. leucotreta* infestation level per orchard/week, based on the full sanitation fruit population. Sampling coverage, thus, uses values within a [0, 1] interval, where values closer to 0 indicate poorer measurement of the true *T. leucotreta* infestation levels and values closer to 1 indicate the better measurement of the true *T. leucotreta* infestation levels. Sampling coverage was treated as being adequate at each sample size, if at least 95% of sub-sample iterations adequately approximated the true population parameter.

Alternatively, the appropriate sample size was determined by using the equation for defining samples size, according to Watson [17], i.e.,

$$n=\frac{\left(\frac{P\left[1-P\right]}{\frac{A^{2}}{Z^{2}}+\frac{P\left[1-P\right]}{N}}\right)}{R}$$

where *n* = sample size required;

*N* = the population size;
*P* = estimated variance in population, as a decimal (e.g., 0.5 for 50–50, 0.3 for 70–30);
*A* = precision desired, expressed as a decimal (i.e., 0.03, 0.05, 0.1 for 3%, 5%, 10%);
*Z* = based on confidence level: 1.96 for 95% confidence, 1.6449 for 90% and 2.5758 for 99%;
*R* = estimated response rate, as a decimal.

The question of orchard size was additionally addressed, and the required sample size for a 1.5 ha orchard and a 20 ha orchard was compared. To achieve this, we assumed a fairly standard density of 505 trees per ha (an orchard spacing of 6.0 m × 3.3 m (rows × trees) would give 505 trees per ha), with a weekly fruit drop of 0.84 fruit per tree per week, which is the population (N). This was the actual average fruit drop per tree per week recorded in the 14 trial orchards used over the two years. The variance (*p*) for the population was unknown, so we made this 0.5. The desired precision level was set as 5%, and thereafter 10%, and the confidence level was 95%. The estimated response rate was 1, i.e., if all inspected fruit were cut, the detectability of infestation should be 100%.

Finally, although not as statistically robust as the Monte Carlo resampling simulation described above for determining mean sample size, possibly a more discerning and less general method for approximating an appropriate sample size from the sanitation fruit was via the visual inspection of the *T. leucotreta* infestation measurements at increasing sample sizes up to total fruit collected across orchards and weeks. Furthermore, this enabled testing whether the fruit sample size determined by the previous two methods could adequately measure *T. leucotreta* infestation in the orchard as a whole. This was carried out using the 2021 data (infestation during 2022 was too low). The number of fruit sampled from the whole orchard sanitation sample was plotted on the *x*-axis, while the *T. leucotreta* infestation level from the whole orchard sanitation sample was plotted on the *y*-axis. An appropriate sample size was then visually determined by comparing the changing level of infestation in the growing sample with that in the sanitation fruit in the orchard as a whole.

#### 2.3.3. Determination of the Need for Random Selection of Samples from Sanitation Fruit from an Orchard for *T. leucotreta* Infestation Determination

To evaluate whether *T. leucotreta* infestation levels (densities) were random or clustered within an orchard, a GLMM framework was applied [13]. *Thaumatotibia leucotreta* densities were modelled per fruit sample from the full sanitation sample as a function of the time before harvest (weeks). The logarithm of the total fruit count per sample lot was specified as an offset variable to account for slight variation in fruit sample sizes (>95% of samples contained either 10 or 20 fruit—this was simply the way in which infestation was recorded). Two competing models were specified, one model assuming a Poisson error distribution and a log-link function, which assumes that *T. leucotreta* densities are randomly distributed amongst the fruit samples (mean = variance), and a second model assuming a negative binomial error distribution and a log-link function, which assumes that *T. leucotreta* densities show a clumped distribution amongst the fruit samples (variance > mean). A Wald’s test (*p* < 0.05) was used to compare the relative support for each model, specifically comparing the log likelihood of each model. If the negative binomial model showed a significantly improved fit over the Poisson model, this would provide strong evidence for a clumped *T. leucotreta* distribution, while if the negative binomial model did not show a significantly improved fit, this would provide evidence for a random *T. leucotreta* distribution. All models were fitted using the ‘glmmTMB’ R package [18].

#### 2.3.4. Determination of Variance in *T. leucotreta* Fruit Infestation per Tree per Week in an Orchard over Time

To evaluate whether *T. leucotreta* infestation levels varied over the growing season, a GLMM approach was used. *Thaumatotibia leucotreta* fruit infestation levels were modelled as a function of time since the start of the growing season (in weeks). Orchard was specified as a random intercept term to account for repeated measurements of *T. leucotreta* infestation from the same orchard over time [13]. The logarithm of the total number of fruit in each sample was specified as an offset in the model formulation to account for variance in fruit sample sizes. The model was specified assuming a negative binomial error distribution and a log link function. The hypothesis of a change in *T. leucotreta* infestation levels over time (weeks) was assessed using a Wald’s test (*p* < 0.05). All modelling was performed in the ‘glmmTMB’ R package [18] and R version 4.4.0 [19].

## 3. Results

Each week during 2021, an average of more than 5000 fruit were collected from the floor of the seven orchards used in the trial (entire orchard plus data trees) and dissected and inspected for *T. leucotreta* infestation. This made up a total of 55,513 fruit for the 11-week duration of the trial (Table 2). Harvesting in the orchards was initiated a week earlier than projected, thus making data collection possible for only 11 of the intended 12 weeks. Furthermore, on two occasions early during the trial (weeks 2 and 4), the farm sanitation team inadvertently removed fruit from underneath data trees, making the data collected during those two weeks superfluous. Consequently, there were 9 weeks of data collection, totalling 63 data collection events (orchard-weeks). Each week during 2022, an average of more than 10,000 fruit were collected from the floor of the seven orchards used in the trial (from entire orchard plus data trees) and dissected and inspected for *T. leucotreta*. This made up a total of 82,958 fruit for the 12-week duration of the trial (Table 2). There are numerous other potential causes of fruit drop, such as environmental and physiological causes, diseases, and other insect pests [20]. However, these were not considered, as they were irrelevant to the purpose of this study.

### 3.1. Thaumatotibia leucotreta Infestation from Data Trees to Measure Infestation in the Orchard as a Whole

In 2021, there was a statistically significant time-varying positive correlation between *T. leucotreta* infestation level measurements from data trees and infestation in the whole orchard (X^2^ = 62.50, df = 1, *p* < 0.001). The correlation decreased slightly over the course of the growing season. However, the full model explained approximately 53.2% of the variation in *T. leucotreta* infestation level measurements from the sanitation fruit from the whole orchard, with more than 50% of the total variation explained by data tree *T. leucotreta* infestation measurements. This result indicates a strong, positive correlation between *T. leucotreta* infestation measurements from data trees and infestation in the whole orchard. Furthermore, in all orchards, infestation from all four sets of data trees combined showed an equivalent or higher level of infestation than the orchard as a whole (Table 3). The average infestation per tree per week for all fruit in the orchard was 0.13, whereas for data trees, this was 0.22. Consequently, the use of data trees overestimated infestation in the orchard as a whole.

Moreover, there was a marginally statistically significant correlation between whether the rate of *T. leucotreta* infestation from data trees was above the selected threshold of 0.1 infested fruit per tree per week versus whether the *T. leucotreta* infestation rate from the total sanitation fruit population was above the 0.1 infested fruit/tree/week (X^2^ = 3.19, df = 1, *p* = 0.074). While not statistically significant per se, the estimated marginal probability of the *T. leucotreta* infestation rate in the whole sanitation sample being above threshold was more than 99.9% likely if the *T. leucotreta* infestation rate in the data tree sample was above threshold, per orchard. None of the orchards that surpassed the infestation threshold of 0.1 infested fruit per tree per week had any set of data trees that did not surpass the threshold, indicating the appropriate sensitivity and reliability of the five-data tree system.

Therefore, although the *T. leucotreta* systems approach states that the most heavily infested section of an orchard must be identified for the placement of the data trees, even regularly positioning a data tree set in the middle of any of the four quarters of an orchard, was sufficient. In 98.41% of cases (data tree sets per week), data were indicative of whether *T. leucotreta* infestation rates were above the 0.1 infested fruit per tree per week threshold in the orchard as a whole (Table 4).

A regression between infestation from the data trees and from the orchard as a whole was not run on the 2022 data, as infestation was only recorded from data trees during one out of 12 weeks in one out of seven orchards. However, as with during 2021, when infestation was recorded from the data trees on this one occasion, infestation was higher than in the orchard as a whole. At no stage during the season was the current systems approach threshold of more than 0.1 infested fruit per tree per week recorded. Infestation was always much lower (Table 5), i.e., 29 to 333 times lower than the threshold. Thus, the recording of zero infestation under the data trees was not problematic, reliably indicating that orchards did not exceed the systems approach threshold.

### 3.2. Sample Size Determination from Sanitation Fruit for Measuring T. leucotreta Infestation in the Total Sanitation Fruit Population from an Orchard

**Figure 2:**
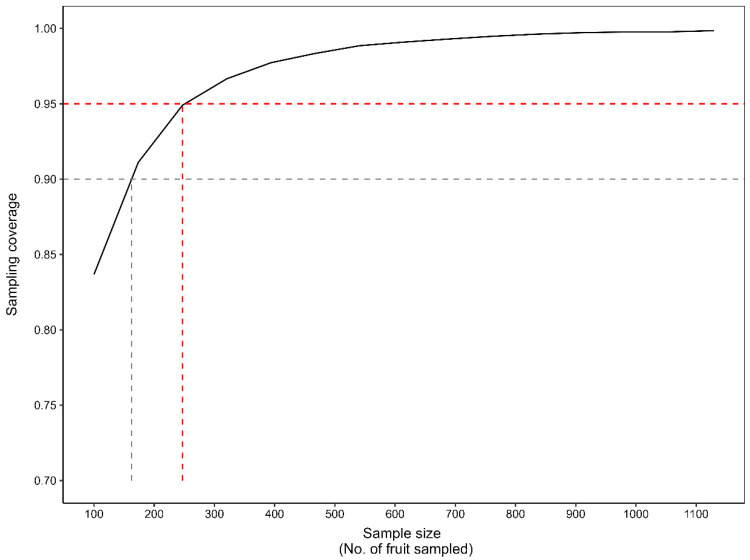
Monte Carlo resampling simulation indicating sampling coverage of various sub-sample sizes to best measure *T. leucotreta* infestation levels from the total sanitation fruit per orchard per week. Sampling coverage indicates the probability of a random sub-sample providing an accurate measurement of the *T. leucotreta* infestation levels from the total sanitation fruit per orchard per week. The red (>95% probability) and grey (>90%) dashed lines indicate possible thresholds for defining sampling coverage adequacy.

The Monte Carlo resampling simulations clearly showed that sampling approximately 245 fruit from the sanitation sample provided sampling coverage values of at least 0.95 (Figure 2, red dashed line). This indicates that, on average, sampling 245 fruit out of the full sanitation sample per orchard would provide an accurate measurement of *T. leucotreta* infestation levels per orchard. However, it is also noticeable that sampling approximately 165 fruit would, on average, provide an accurate measurement of *T. leucotreta* infestation levels per orchard in more than 90% of the samples taken (Figure 2, grey dashed line).

Using the formula of Watson [17], sample size determined for a 1.5 ha orchard was 240 fruit. This is not dissimilar to the sample size determined from the Monte Carlo resampling simulation of 245 for the orchards used, which were also all around 1.5 ha in size. However, for a very large orchard, e.g., 20 ha, sample size determined according to Watson [17] was 368. In the southern African citrus industry, more than 99% of orchards are smaller than this (Citrus Growers’ Association, unpublished data), and the required sample size would also increase little for larger orchards. If the precision desired (A), or margin of error, is reduced from 5% to 10%, the calculated sample size becomes 78 and 95 for orchards of 1.5 ha and 20 ha, respectively. This can conveniently be rounded up to a sample of 100 fruit. A precision of 5% would mean that if a sample shows an extrapolated infestation of 0.1 infested fruit per tree per week, in reality it could range between 0.095 and 0.105 in the population (orchard), whereas a precision of 10% would imply a range of 0.09 to 0.11 infested fruit per tree per week in the whole orchard.

Lastly, an appropriate sample size for measuring *T. leucotreta* infestation from the sanitation fruit from the whole orchard, was assessed via the visual inspection of the *T. leucotreta* infestation levels at various sample sizes across orchards and weeks using the 2021 data. This also enabled the determination of whether the 100-fruit sample size, from the previous calculation, adequately and reliably measured infestation in the orchard as a whole. To do so, the number of fruit sampled from the whole orchard sanitation sample was plotted on the *x*-axis, while the *T. leucotreta* infestation level from the whole orchard sanitation sample was plotted on the *y*-axis.

Across all weeks and orchards in 2021, a sample of approximately 100 fruit from the whole orchard sanitation fruit population was indeed sufficient to provide a reliable measurement of the *T. leucotreta* infestation from the whole orchard (Figure S1). Although infestation in the first 100 fruit sample was higher than or equivalent to infestation in the sanitation fruit from the whole orchard in only 54 out of 63 orchard-weeks (85.7%), where infestation in the whole population of sanitation fruit exceeded the threshold of 0.1 infested fruit per tree per week, this threshold was also exceeded in all (100%) of the first 100-fruit samples from the sanitation fruit. As such, a subsample of 100 fruit per orchard per week from the whole orchard sanitation sample would provide a reliable measurement of *T. leucotreta* infestation levels in the orchard as a whole and an even better measurement of threshold exceedance in the orchard as a whole. As the 2022 data consisted mainly of nil values for *T. leucotreta* infestation, the same calculations could not be conducted with these data as was performed with the 2021 data.

### 3.3. Determination of the Need for Random Selection of Samples from Sanitation Fruit from an Ochard for T. leucotreta Infestation Determination

**Figure 3:**
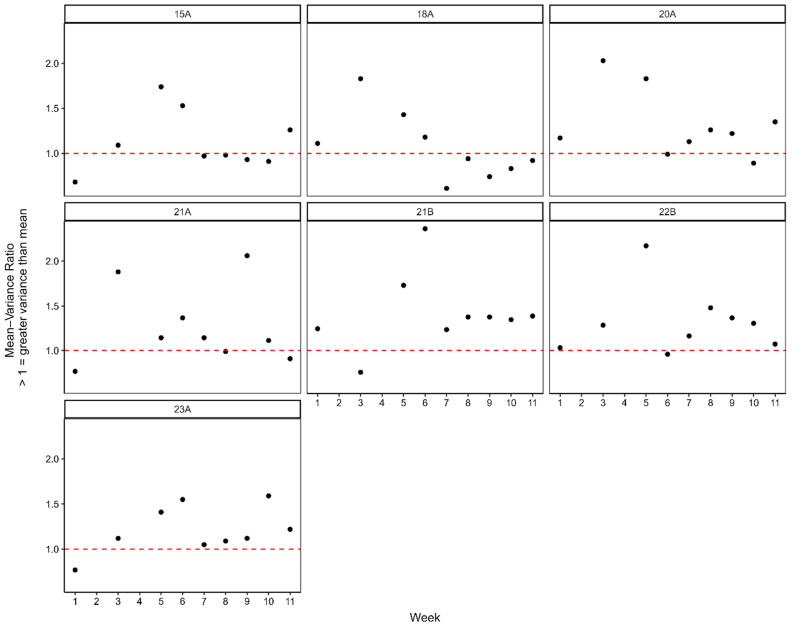
Mean–variance relationships showing the distribution of *T. leucotreta* within sanitation samples for each of the seven orchards monitored during 2021. Mean–variance values approximately equal to 1 (indicated by the red dashed line) are indicative of randomly dispersed *T. leucotreta* counts across samples, while values greater than 1 are indicative of increasingly clumped counts across samples.

Over the 11 weeks of monitoring during 2021, there was some evidence for the spatial clumping of *T. leucotreta* infestation levels amongst fruit samples (Figure 3 and Table 5). However, the magnitude of spatial clumping was variable between orchards, with some orchards (e.g., 4) showing no evidence of the spatial clumping of *T. leucotreta* infestation levels (Table 6). Additionally, averaged across orchards and over the growing season, the magnitude of spatial clustering was relatively low, with the vast majority of week/orchard mean-variance ratios < 2 (Figure 3).

Moreover, visualising the distribution of spatial clustering over time clearly indicated that the degree of spatial clustering decreased over time, becoming more random closer to harvest (Figure 3). When we investigated the distribution of *T. leucotreta* within the sanitation fruit samples in only the last four weeks prior to harvest (week 8–11), there was strong support for *T. leucotreta* infestation levels being randomly dispersed (Table 6). Six out of the seven orchards showed support for randomly dispersed *T. leucotreta* infestation levels, with only orchard 7 showing support for a slightly clumped *T. leucotreta* distribution in the sanitation samples (Figure 3).

**Figure 4:**
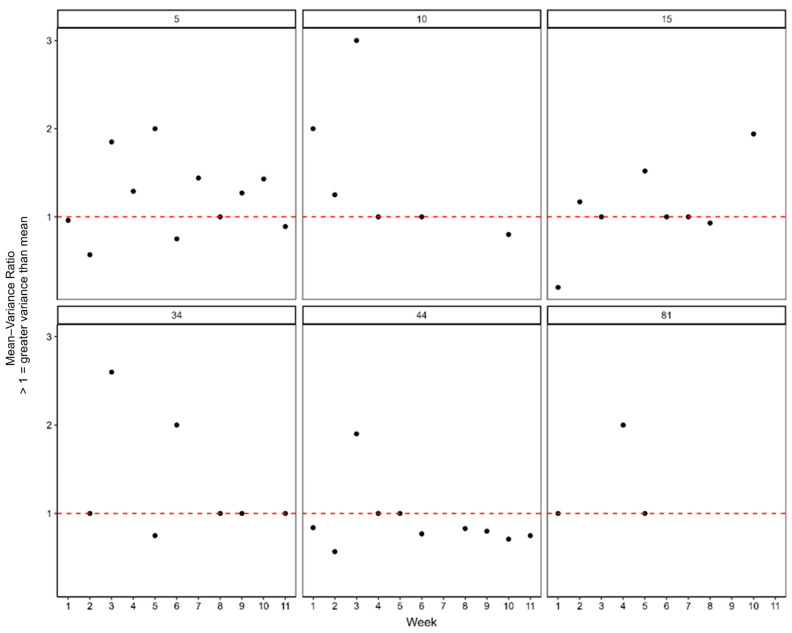
General linear model showing the distribution of *T. leucotreta* within sanitation samples for each of six orchards monitored during 2022. The seventh orchard (orchard 19) was excluded, as only one infested fruit was recorded in the orchard during the 12-week period. Mean–variance values approximately equal to 1 (indicated by the red dashed line) are indicative of randomly dispersed *T. leucotreta* counts across samples, while values greater than 1 are indicative of increasingly clumped counts across samples.

Over the 12 weeks of monitoring during 2022, there was some evidence for the spatial clumping of *T. leucotreta* infestation levels amongst fruit samples (Figure 4 and Table 7). However, the magnitude of spatial clumping was variable between orchards, with some orchards (e.g., 5, 15, 44) showing no evidence of the spatial clumping of *T. leucotreta* infestation levels (Table 6). Additionally, averaged across orchards and over the growing season, the magnitude of spatial clustering was relatively low, with the vast majority of week/orchard mean–variance ratios < 2 (Figure 4).

### 3.4. Determination of Variance in T. leucotreta Fruit Infestation per Tree per Week in an Orchard over Time

**Figure 5:**
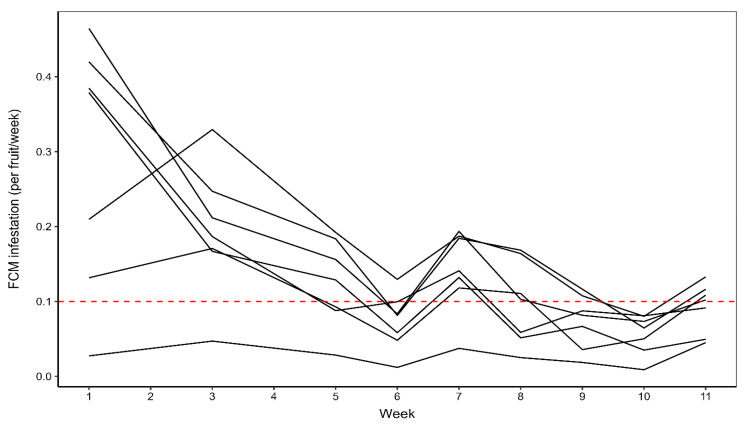
*Thaumatotibia leucotreta* fruit infestation per tree per week for the seven orchards monitored during the 2021 growing season. The solid black lines indicate mean *T. leucotreta* infestation per tree per week, per orchard. The red dashed line indicates the *T. leucotreta* fruit infestation exceedance threshold of 0.1 infested fruit per tree per week.

Over the 11 weeks of monitoring during 2021, there was evidence for a statistically significant reduction in *T. leucotreta* infestation per tree per week (X^2^ = 783.77, df = 1, *p* < 0.001). There was a mean measured decrease in *T. leucotreta* infestation per tree per week of 0.02 ± 0.01 units per each additional week, averaged across orchards and the growing season (Figure 5). This implies that there was not a dramatic change in the level of *T. leucotreta* infestation in most of the orchards. This indicates that if the weekly monitoring of fruit infestation is too laboriously onerous, fortnightly assessments would suffice, with little risk of altered infestation levels and thus little risk of loss of critical data.

It should be noted, however, that there was a small increase in infestation in the final week (week 11), with three out of the seven orchards (20A, 22B and 23A) exceeding the threshold of 0.1 *T. leucotreta* fruit infestation/tree/week (Figure 5). At least two monitoring events, two weeks apart, would therefore be necessary and adequate during these last four weeks before harvest, preferably with the last monitoring event during the final week before harvest.

**Figure 6:**
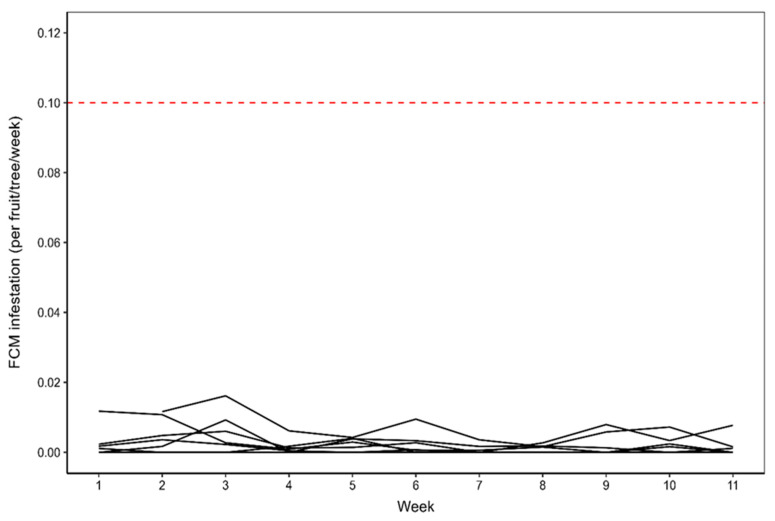
*Thaumatotibia leucotreta* fruit infestation per tree per week for the seven orchards monitored during the 2022 growing season. The solid black lines indicate mean *T. leucotreta* infestation per tree per week, per orchard. Infestation was consistently below the *T. leucotreta* fruit infestation exceedance threshold of 0.1 infested fruit per tree per week (indicated by the red horizontal line).

Over the 12 weeks of monitoring during 2022, there was evidence for a statistically significant reduction in *T. leucotreta* infestation per tree per week (X^2^ = 37.56, df = 1, *p* < 0.001). There was a mean measured decrease in *T. leucotreta* infestation per tree per week of 13% per each additional week, averaged across orchards and the growing season (Figure 6). The observed decrease in *T. leucotreta* infestation was most notable after the third week of monitoring, with a slight mean increase in infestation during the last four weeks before harvest, relative to the preceding four weeks, but this was not statistically significantly different (X^2^ = 0.10, df = 1, *p* = 0.752) (Figure 6). Additionally, infestation was extremely low throughout, and it is thus unlikely that these small changes in a very low level of infestation have any meaning.

## 4. Discussion

The primary objective of this study was to evaluate the efficacy of preharvest monitoring procedures for *T. leucotreta* infestation in citrus, for inclusion in a systems approach for export risk mitigation. The appropriateness and usefulness of the measurement of pest level, through sampling, were determined according to their ability to statistically estimate the pest population in the orchard as a whole. Over two citrus producing seasons, comprehensive and extensive datasets were collected. Crucially, these data represented direct measurements of *T. leucotreta* infestation from all fallen fruit during each monitoring period, rather than relying on samples. This approach provided comprehensive and complete data, given that all *T. leucotreta* infested fruit are known to drop from the tree, against which the monitoring procedures could be compared [11,12]. Consequently, in evaluating the preharvest infestation monitoring procedures for *T. leucotreta*, an absolute, rather than estimated benchmark was available.

In determining if *T. leucotreta* infestation in fruit fallen from data trees was an accurate measurement of infestation in the orchard as a whole, there was a significant positive correlation between *T. leucotreta* infestation from the five data tree sets and infestation in the whole orchard. In 2021 average infestation from the data tree sets (both per orchard and across all orchards) was always higher than from the orchard as a whole. Overall average infestation from data tree sets was 0.24 infested fruit per tree per week, compared with the 0.13 infested fruit per tree per week from the orchard as a whole. Although in season one, fewer than 50% of five-data-tree sets per week showed the same or higher infestation than in the orchard as a whole, in 82.54% of orchard-weeks, at least one five-data-tree set per orchard had an infestation level equal to or higher than the orchard’s overall infestation. Where infestation in the orchard as a whole exceeded the threshold of 0.1 infested fruit per tree per week, fewer than 2% of data tree sets did not show this. During any week when infestation in the orchard exceeded the threshold, at least one five-data-tree set in that orchard also indicated the threshold exceedance. This indicates that the five-data-tree system is effective in measuring *T. leucotreta* infestation in the orchard and even more effective as a measure of infestation threshold exceedance in the orchard. The *T. leucotreta* systems approach indicated that the five-data-tree set should be positioned at a place in the orchard where fruit drop (and thus presumably *T. leucotreta* infestation) is highest, with the objective of ensuring that the data trees are well positioned to effectively (or even excessively) measure *T. leucotreta* infestation in the orchard as a whole. From the 2021 data, it was evident that even if the data trees are randomly positioned the measurement is effective, since the failure of the five data trees to indicate threshold exceedance in the orchard was less than 2%.

This study indicated that *T. leucotreta* infestation within an orchard as a whole does not show a strongly clumped distribution. However, the five-data-tree system measured higher *T. leucotreta* infestation than in the orchard as a whole. The data trees were positioned in the centre of four quadrants, meaning that there were several rows of citrus trees between these data trees and the orchard border, protecting the data trees from wind. The Eastern Cape, where the trials were conducted, is notoriously windy and, consequently, the planting of windbreaks around orchards is a recommended and standard practice [21,22]. It has been reported that *T. leucotreta* egg laying is higher in localities with wind protection, seemingly due to gravid female *T. leucotreta* adults using the protection of windbreaks for undisturbed oviposition [23]. The centres of orchards may be more wind-protected, leading to higher *T. leucotreta* infestation in orchard centres than in tree rows closer to the perimeter of the orchard.

Whereas this study showed that the five-data-tree system for preharvest *T. leucotreta* infestation monitoring can be an effective measurement procedure, under commercial farming conditions, the system is vulnerable to disturbance through routine orchard sanitation practices. Orchard sanitation is one of the most important and effective practices in suppression of *T. leucotreta* in citrus orchards. Weekly sanitation, through the removal and destruction of fallen fruit, has been shown to reduce the *T. leucotreta* population in an orchard by an average of 75% [24]. This was concluded through the analysis of data collected over a 10-year period, and if sanitation is conducted more frequently than once per week and if apparently infested and damaged hanging fruit are removed in addition to all fallen fruit, the efficacy of the practice exceeds 75%. Consequently, orchard sanitation is a mandatory practice within the *T. leucotreta* systems approach. Most citrus farms in South Africa have full time sanitation teams, which go from orchard to orchard, removing hanging fruit that appear to be infested or damaged, and all fallen fruit. Although data trees, used for the preharvest monitoring of *T. leucotreta* infestation, are generally marked in some manner, and sanitation teams are instructed not to collect fruit from underneath and on these trees, there remains risk that the strict exclusion of data trees is not always achieved. This provides strong justification for applying an alternative preharvest infestation monitoring system that is less prone to such unintended interference.

The alternative system investigated in this study entails regularly analysing a sample of fruit drawn from all the fruit collected during orchard sanitation, instead of using data trees. In developing such a system, this study investigated the fruit sample size required, the need for random selection of the sample and required frequency of sampling.

To determine sample size, by conducting a Monte Carlo simulation with almost 10,000 iterations, it was determined that by using a sample size of 245 fruit, 95% of such samples provide an accurate measurement (above the lower bound of the 95% confidence interval) of the true *T. leucotreta* larval infestation of fruit in the orchard as a whole [16]. By using a sample size of 165 fruit, 90% of such samples provide an accurate approximation of the *T. leucotreta* larval infestation of fruit in the orchard as a whole. Alternatively, by using a standard formula for sample size determination, a sample of 384 sanitation fruit from an orchard was required for a 5% level of precision, with 95% confidence, whereas a sample of 96 fruit was required for a 10% level of precision. This was conservatively determined for a very large orchard, i.e., 20 ha. A citrus orchard of such a size is not usual in the southern African citrus industry, with 99% of orchards being smaller. A potential criticism of using a standard sample size formula is that the variance in the population is unknown and that one therefore has to assume a homogenous population distribution in the orchard. However, this study did determine that this is a reasonable assumption, indicating that *T. leucotreta* infestation is fairly homogenously distributed in various fruit samples taken from throughout an orchard.

We consider the 165 (90% confidence level) to 245 (95% confidence level) fruit sample size approximated by the Monte Carlo resampling simulation to be unnecessary and excessive for the following reasons. Firstly, if one was taking a single sample from an orchard to reliably measure *T. leucotreta* infestation in the orchard as a whole, it would be advisable to take such a large sample of sanitation fruit. However, the infestation monitoring procedure in the systems approach entails multiple samples taken over at least 12 weeks. A failure to detect threshold exceedance during one sampling is highly unlikely to reoccur in the following sampling, at which time the necessary corrective action can be taken. The systems approach entails fortnightly sampling, so at least six samples will be taken over this 12-week period. The visual inspection of the *T. leucotreta* infestation levels with various sample sizes across orchards and weeks using the 2021 data, demonstrated that a 100-fruit sample size is a reliable measure of infestation in the orchard as a whole. The exceedance of the 0.1 infested fruit per tree per week threshold was always also evident in the inspection of the first 100 fruit sampled—a 100% successful prediction rate. Furthermore, in our study, samples were not taken randomly; they were inspected and dissected in the order that they were collected from the different parts of an orchard. If samples were taken randomly, any one sample may have been more representative of the whole orchard. Non-random sampling could potentially lead to a slight under or over estimation. Consequently, the recommendation of random sampling is prudent. Nonetheless, the results confirm that the 100-fruit sample size (rounded up from 95), as calculated according to a standard sample size calculation formula, using a 10% precision rate and a 95% confidence limit, provides an appropriate and reliable sample size.

The average size of a citrus farm in South Africa is 58 ha, with the average orchard size being 3.88 ha (Citrus Growers’ Association, unpublished data). Consequently, on each occasion that a preharvest infestation analysis is conducted on-farm, using a 100-fruit sample size, on average 1500 fruit need to be individually dissected and inspected. On the upper end, a very large farm could be in excess of 1000 ha, with an average orchard size of 11.18 ha, requiring almost 13,000 fruit to be dissected and inspected on each occasion. A 100-fruit sample size has been demonstrated to enable the effective measurement of *T. leucotreta* infestation in an orchard as a whole, even for very large orchards, indicating that a larger fruit sample size is unnecessary.

The random selection of fruit during sampling is recommended in the systems approach procedures. In this study, fruit were not analysed (dissected and inspected) in random order. Fruit were collected in crates in the order in which they were collected from the different parts of the orchard. Analyses were then conducted crate by crate, thus offering the opportunity to determine whether the distribution of *T. leucotreta* infestation throughout an orchard was clumped or random. Until the last four weeks before harvest, infestation showed a low level of spatial clumping in all but one of the orchards. This disappeared in all but one of the orchards during the last four weeks before harvest, with infestation throughout orchards becoming randomly distributed. This indicated that if samples are not randomly selected from the population of sanitation fruit from an orchard on each occasion, the measurement will not be adversely affected, especially during the last four weeks before harvest, when the accuracy of sampling in the systems approach is most important. This was also reflected in the finding that in all cases where infestation in the orchard exceeded the threshold of 0.1 infested fruit per tree per week, this was also exceeded in the first 100 fruit sample inspected. In the second season (2022), infestation within the orchard was found to be more clumped in nature. However, with the extremely low level of infestation recorded in these orchards, where the threshold of 0.1 infested fruit per tree per week was never reached (always a minimum of 33 times lower), this stands to reason and this slightly clumped distribution has no relevance in terms of how sample selection may have affected the detection of potential threshold exceedance.

The final question that this study addressed was that of variance in *T. leucotreta* infestation over time, leading up to harvest. In both seasons, there were no dramatic changes in the level of *T. leucotreta* infestation in most of the orchards over the 11–12-week period of monitoring. However, a slight mean decrease in *T. leucotreta* infestation was observed over this time.

In the first season, the highest mean recorded infestation was in the first week of monitoring, whereas in the second season, this occurred in the third week of monitoring, albeit at a much lower level than was the case with the orchards during the first season. This indicates that if the weekly monitoring of fruit infestation is impractical, fortnightly assessments would suffice, without risking the loss of reliability in measured infestation levels.

In 2021, there was a small increase in infestation in the final week (week 11), with three out of the seven orchards exceeding the threshold of 0.1 *T. leucotreta* fruit infested per tree per week and another equalling the threshold. However, all of these orchards had exceeded the threshold previously in the season, to a greater extent than during this last week before harvest. With this forewarning, it was thus no surprise that there was another threshold exceedance. In the second season, there was a slight mean increase in infestation during the last four weeks before harvest, relative to the previous four weeks, but this was not significant, and infestation was still dramatically lower than the threshold. Nonetheless, it would be advisable for there to be at least two monitoring events during these last four weeks before harvest, ideally with the last one during the last week before harvest. Although this study involved a limited number of orchards (14) over two seasons, it is highly unlikely that any citrus orchards will experience a dramatic spike in *T. leucotreta* infestation in the weeks leading up to harvest, as *T. leucotreta* is a very slow disperser [11,25,26,27]. Thus, *T. leucotreta* population levels in adjacent orchards and natural vegetation will have a negligible effect in the short to medium term [11,28]. Furthermore, good *T. leucotreta* management is now practised throughout the southern African citrus industry, with most orchards being prepared for potential export to markets sensitive to *T. leucotreta* infestation, precluding the tolerance of any moderate to high pest populations [3,29,30,31].

### Benefits of and Recommendations for the New System

The new preharvest infestation monitoring system, which utilises a 100-fruit sample drawn directly from orchard sanitation collections, offers meaningful advantages for robust *T. leucotreta* management. This approach inherently enhances reliability by circumventing the risk of inadvertent data loss or interference that can occur with marked data trees during routine orchard sanitation practices. Our findings demonstrate that this 100-fruit sample is accurate and sensitive, consistently detecting when orchard-wide infestation exceeds the threshold of 0.1 infested fruit per tree per week. Furthermore, this sample size is practical and efficient for commercial application, providing effective measurement even for very large orchards, without necessitating excessive fruit dissection.

The employment of this monitoring system, using a threshold of 0.1 infested fruit per tree per week, was found to be an effective system for incorporation into the systems approach as a more robust alternative to the five-data-tree monitoring system. It ensures that only fruit with no more than an extremely low level of *T. leucotreta* infestation are delivered to the packing house. All infested fruit will drop off the tree [11,12]. This will occur within an estimated 4 weeks after infestation occurs. In a worst-case scenario, if this threshold is reached every week leading up to harvest, a 0.1 infested fruit per tree per week recorded in dropped fruit, would mean 0.3 infested fruit per tree remaining on the tree. Therefore, in a crop of 45 tonnes per ha (which was the average yield of all orchards used for this study in both years), at an average fruit size of 72 fruit per 15 kg carton, with 555 trees per ha (i.e., accepted general averages), infestation in fruit at harvest would total around 0.08%. Note that this is indeed a worst-case scenario and that in reality, infestation is far lower than this (PhytClean, unpublished data). Furthermore, the systems approach includes in-orchard fruit culling. Fruit showing signs of potential *T. leucotreta* infestation must be removed during the picking process within the orchard and excluded from packing for export. This would further reduce the maximum possible infestation of fruit delivered to the packing house.

The expression of pest infestation as infested fruit per tree per week provides a reliable estimate of pest population density and is independent of crop size (fruit per tree or per unit area), particularly as the host resource (fruit) way exceeds what is required by the pest. However, this metric, and the threshold of 0.1 infested fruit per tree per week, does not speak to the proportion of fruit infested, as this is indeed dependent on crop size. Nonetheless, this threshold is so low that even in an orchard where crop load is much lower than the 45 tonnes per ha (an estimated 389 fruit per tree), the proportion of fruit infested would still be extremely low. Furthermore, this measurement is not used in the calculation of the efficacy of the systems approach but simply ensures that only fruit from orchards with an extremely low *T. leucotreta* population density will be considered for export under the systems approach. Immediately on the delivery of fruit from an orchard to the packinghouse, a representative sample of fruit is taken and inspected for infestation, providing the necessary data on infestation level of fruit from the orchard, with associated thresholds for continued participation for export within the systems approach [6].

Infestation recorded in the orchard can be influenced by management practices, such as orchard sanitation, pesticide sprays, mating disruption, and the sterile insect technique [3]. Furthermore, different citrus types and cultivars vary in their susceptibility to *T. leucotreta* and *T. leucotreta* pest pressure varies across the different commercial citrus production regions in South Africa [5]. Consequently, one can expect differences in infestation between orchards. However, these factors are inconsequential to the efficacy and reliability of the infestation monitoring system, as *T. leucotreta* population density is accurately measured, regardless of the factors that may influence this.

Considering the observed temporal homogeneity of *T. leucotreta* infestation over the monitoring period, fortnightly assessments are recommended as sufficient, thereby optimising resource allocation without compromising the detection of potential infestation spikes. While a random selection of fruit within the sanitation sample is generally advised to account for minor spatial clumping, our study showed that non-random sampling did not negatively impact measurement sensitivity, particularly in the crucial last four weeks before harvest when infestation distribution tends to become more uniform. Ultimately, this system provides a practical, reliable, and integrated tool that strengthens phytosanitary risk mitigation, ensuring that only fruit with extremely low *T. leucotreta* infestation levels proceed to the packing house.

## 5. Conclusions

A systems approach has been commercially implemented since 2017 for the management and phytosanitary risk mitigation of *T. leucotreta* in fresh citrus fruit exported from South Africa [5,6]. The system has been demonstrated to be at least as efficacious as the Probit 9 standard associated with certain standalone postharvest disinfestation treatments [6]. An important component of the first measure in this three-measure systems approach, is preharvest fruit infestation monitoring. At the inception of the system, a system employing groups of five data trees per orchard, with a sliding scale for further sets of data trees, based on orchard size, was used. This study validated the use of this data tree monitoring system, by showing that on average, it overestimated the level of *T. leucotreta* infestation in the orchard as a whole. Furthermore, where infestation in the orchard as a whole exceeded a selected threshold of 0.1 infested fruit per tree per week, the threshold was also exceeded in more than 98% of orchard-weeks during monitoring. Despite the proven efficacy of the system, there is a risk that farm workers conducting orchard sanitation in orchards, may inadvertently remove fruit fallen underneath these data trees, before monitors can collect the fruit for infestation analysis. Consequently, a new preharvest infestation monitoring system was developed as a more robust alternative.

The new system avoids the risk of inadvertent loss of data that could be associated with the data tree system, as it draws samples from all the fruit collected during orchard sanitation. Our study determined that a 100-fruit sample taken from the sanitation fruit and dissected to inspect for *T. leucotreta* infestation, would be adequate to accurately measure infestation in the orchard as a whole.

In conclusion, a preharvest infestation monitoring system, involving the inspection of a randomly selected 100-fruit sample of the total sanitation fruit per orchard each fortnight, for several weeks leading up to harvest, is suitable for application in the *T. leucotreta* systems approach used for export of fresh citrus in South Africa.

## Figures and Tables

**Table 1:** Details of Navel orange orchards in the Sundays River Valley, Eastern Cape, evaluated in 2021 and 2022.

Data Year	Orchard Number	Variety	Year Planted	Area (ha)	No. of Trees per Orchard	Co-Ordinates	
2021	15A	Lane late	2003	1.33	741	33°27′45.96″ S	25°34′6.33″ E
	18A	Cambria	2003	1.46	810	33°27′36.17″ S	25°34′18.58″ E
	20A	Cambria	2003	1.30	720	33°27′55.46″ S	25°34′8.89″ E
	21A	Powell	2003	1.46	813	33°27′52.31″ S	25°34′13.23″ E
	21B	Powell	2003	1.47	803	33°27′55.08″ S	25°34′16.48″ E
	22B	Powell	2003	1.46	809	33°27′51.71″ S	25°34′20.93″ E
	23A	Autumn Gold	2003	1.48	823	33°27′45.28″ S	25°34′21.56″ E
2022	5	Autumn Gold	2010	2.58	1435	33°32′5.26″ S	25°39′26.79″ E
	10	Autumn Gold	2010	2.09	1160	33°31′40.69″ S	25°39′41.35″ E
	15	Autumn Gold	2010	2.51	1393	33°31′53.73″ S	25°39′37.79″ E
	19	Midnight	1996	2.58	678	33°32′1.64″ S	25°39′35.47″ E
	34	Autumn Gold	2010	2.28	1432	33°31′47.56″ S	25°39′40.38″ E
	44	Witkrans	2009	1.02	1296	33°32′3.51″ S	25°39′14.77″ E
	81	Midnight	2003	2.05	1137	33°32′3.19″ S	25°39′37.65″ E

**Table 2:** Total number of fruit collected and dissected from each orchard (four sets of five data trees per orchard plus total orchard) over the 9- and 12-week periods for 2021 and 2022, respectively.

Year	Orchard	Fruit from Data Trees				Total Fruit Collectedand Dissected from:		Average Number of Fruit per Data Tree per Week	Average Number of Fruit per Orchard Tree per Week
		A	B	C	D	All Data Trees	Whole Orchard		
2021	15A	42	83	47	52	224	10,222	1.24	1.53
	18A	23	89	27	16	105	5113	0.58	0.70
	20A	61	38	60	36	197	6302	1.09	0.59
	21A	14	5	25	58	102	3948	0.57	0.54
	21B	35	10	50	36	135	4713	0.75	0.65
	22B	61	57	58	96	283	7461	1.57	1.03
	23A	67	82	83	37	273	10,735	1.51	1.43
	Total					1530	53,983	1.21	1.09
2022	5	96	38	41	32	223	10,670	0.93	0.62
	10	39	148	227	101	515	17,573	2.14	1.26
	15	99	20	29	102	266	20,066	1.48	1.2
	19	4	6	6	7	28	2128	0.12	0.26
	34	203	78	25	56	399	8713	1.67	0.51
	44	131	117	112	101	515	17,573	2.14	1.26
	81	11	44	27	0	89	8609	0.37	0.63
	Total					1989	80,969	1.18	0.79

**Table 3:** *Thaumatotibia leucotreta* infested fallen fruit per tree per week for each data set in each orchard, all data sets per orchard and total fallen fruit in each orchard, during 2021. Data in bold is where infestation is equivalent to or higher than in the whole orchard. Data in italics indicates exceedance of a threshold of 0.1 infested fruit per tree per week.

Orchard	Infested Fruit per Tree per Week					
	Set of Five Data Trees				All Sets of Data Trees	Whole Orchard
	A	B	C	D		
15A	***0.20***	***0.42***	*0.11*	***0.33***	***0.26***	*0.15*
18A	0.00	**0.05**	**0.02**	0.00	**0.02**	0.02
20A	***0.27***	***0.22***	*0.13*	***0.16***	***0.21***	*0.17*
21A	0.04	0.00	**0.11**	**0.29**	**0.11**	0.06
21B	**0.25**	0.02	**0.16**	**0.11**	**0.15**	0.09
22B	***0.40***	***0.38***	***0.22***	***0.58***	***0.45***	*0.18*
23A	***0.29***	***0.65***	***0.64***	***0.22***	***0.48***	*0.21*

**Table 4:** Summarised comparative data between *T. leucotreta* fruit infestation from five-data-tree sets (four per orchard) over all orchards (seven) and weeks (nine) (i.e., 252 “cases”), relative to infestation in sanitation from the whole orchard, for the 2021 season.

Cases that showed infestation higher or equal to that in the orchard as a whole.	47.62%
Cases where none of the four data tree sets in an orchard showed infestation equal to or higher than in the orchard as a whole.	17.46%
Cases where data tree sets did not show threshold exceedance (>0.1 infested fruit/tree/week), when threshold was exceeded in whole orchard.	1.59%
Cases where no five-data-tree sets showed threshold exceedance, when threshold was exceeded in the whole orchard.	0%

**Table 5:** *Thaumatotibia leucotreta* infested fallen fruit per tree per week for each data set in each orchard, all data sets per orchard and total fallen fruit in each orchard, during 2022.

Orchard	Infested Fruit per Tree per Week					
	Set of Five Data Trees				All Sets of Data Trees	Whole Orchard
	A	B	C	D		
10	0	0	0	0	0	0.0010
44	0	0	0	0	0	0.0030
15	0	0	0	0	0	0.0018
34	0	0	0	0	0	0.0009
5	0	0	0	0.017	0.004	0.0035
81	0	0	0	0	0	0.0003
19	0	0	0	0	0	0.0001

**Table 6:** Wald’s test supporting the GLMM of the distribution of *T. leucotreta* within sanitation samples from the seven orchards monitored during 2021.

Orchard	Time Period							
	All Weeks				Week 8–11			
	Distribution	X^2^	df	*p*	Distribution	X^2^	df	*p*
15A	Clumped	19.31	1	<0.001	Random	0.26	1	0.607
18A	Clumped	9.45	1	0.002	Random	0	1	1
20A	Clumped	11.10	1	<0.001	Random	0.29	1	0.587
21A	Random	2.53	1	0.117	Random	0.37	1	0.542
21B	Clumped	5.56	1	0.018	Random	1.97	1	0.156
22B	Clumped	6.46	1	0.011	Random	0.59	1	0.444
23A	Clumped	6.03	1	0.014	Clumped	5.36	1	0.021

**Table 7:** Wald’s test supporting the GLMM of the distribution of *T. leucotreta* within sanitation samples from six orchards monitored during 2022. The seventh orchard (orchard 19) was excluded, as only one infested fruit was recorded in the orchard during the 12-week period.

Orchard	All Weeks			
	Distribution	X^2^	df	*p*
5	Random	1.74	1	0.187
10	Clumped	7.47	1	0.006
15	Random	0.01	1	0.929
34	Clumped	6.75	1	0.009
44	Random	0.01	1	0.893
81	Random	3.09	1	0.082

## Data Availability

The original contributions presented in this study are included in the article/Supplementary Materials. Further inquiries can be directed to the corresponding author.

## Supplementary Materials

The following supporting information can be downloaded at: https://www.mdpi.com/article/10.3390/insects16060589/s1, Figure S1a–g. Sample size assessment required to provide a reliable measurement of *T. leucotreta* infestation in the sanitation fruit population from the whole orchard for orchards 1 (a), 2 (b), 3 (c), 4 (d), 5 (e), 6 (f), and 7 (g) in 2021. The red line indicates the *T. leucotreta* infestation level in the sanitation fruit population from the whole orchard. The black line indicates *T. leucotreta* infestation level in the subsample of sanitation fruit from the orchard. The vertical green line indicates the first 100 fruit sample, and the horizontal green line indicates the threshold of 0.1 infested fruit per tree per week. The *T. leucotreta* estimate provided by the subsample provided a reliable measurement of the whole orchard infestation level wherever the black line is equivalent to or higher than the red line. The panels indicate different weeks (weeks 1–11, excluding weeks 2 and 4).

## Abbreviations

The following abbreviations are used in this manuscript:

FCM	False Codling Moth
EU	European Union
ISPM	International Standards for Phytosanitary Measures
IPPC	International Plant Protection Convention
GLMM	Generalised Linear Mixed Modelling
