# Supplementary material for: Development and Evaluation of Preharvest Thaumatotibia leucotreta Citrus Fruit Infestation Monitoring for Inclusion in a Systems Approach
Source: Insects. 2025 Jun 3;16(6):589. doi: 10.3390/insects16060589 (PMC12192775; doi:10.3390/insects16060589)
Supplement: Supplementary file 1 [file insects-16-00589-s001.zip › insects-3664939-supplementary.pdf]

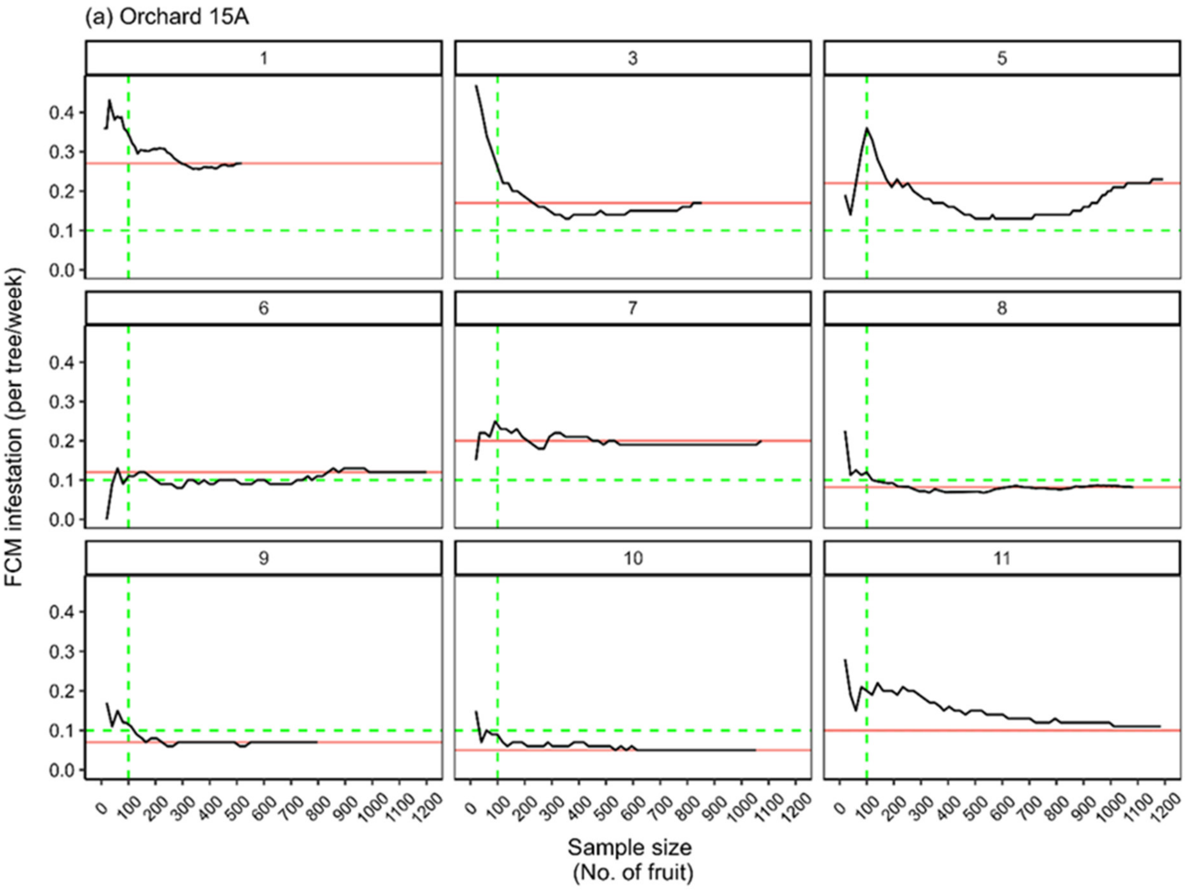
(a) Orchard 15A
1
3
5
6
7
8
9
10
11
FCM infestation (per tree/week)
0.0
0.1
0.2
0.3
0.4
0
100
200
300
400
500
600
700
800
900
1000
1100
1200
Sample size
(No. of fruit)

(b) Orchard 18A

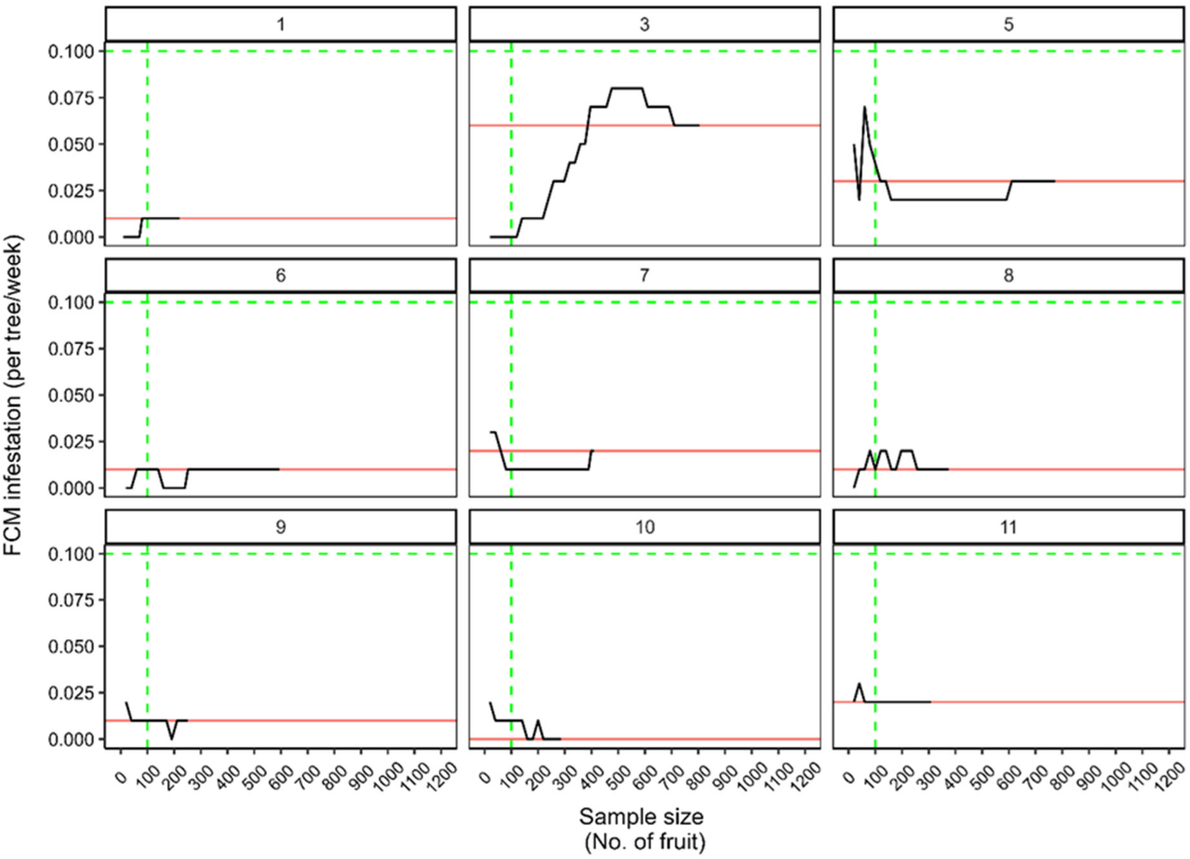

(c) Orchard 20A

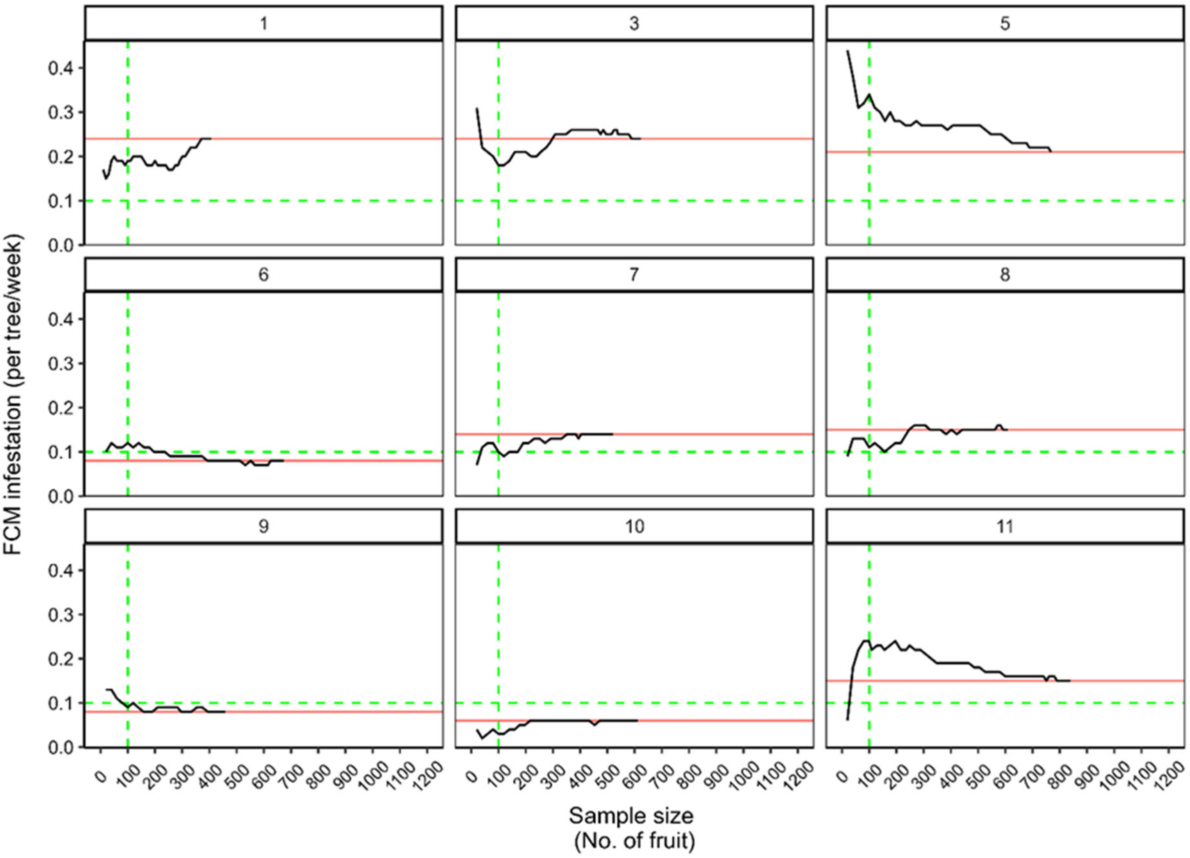

(d) Orchard 21A

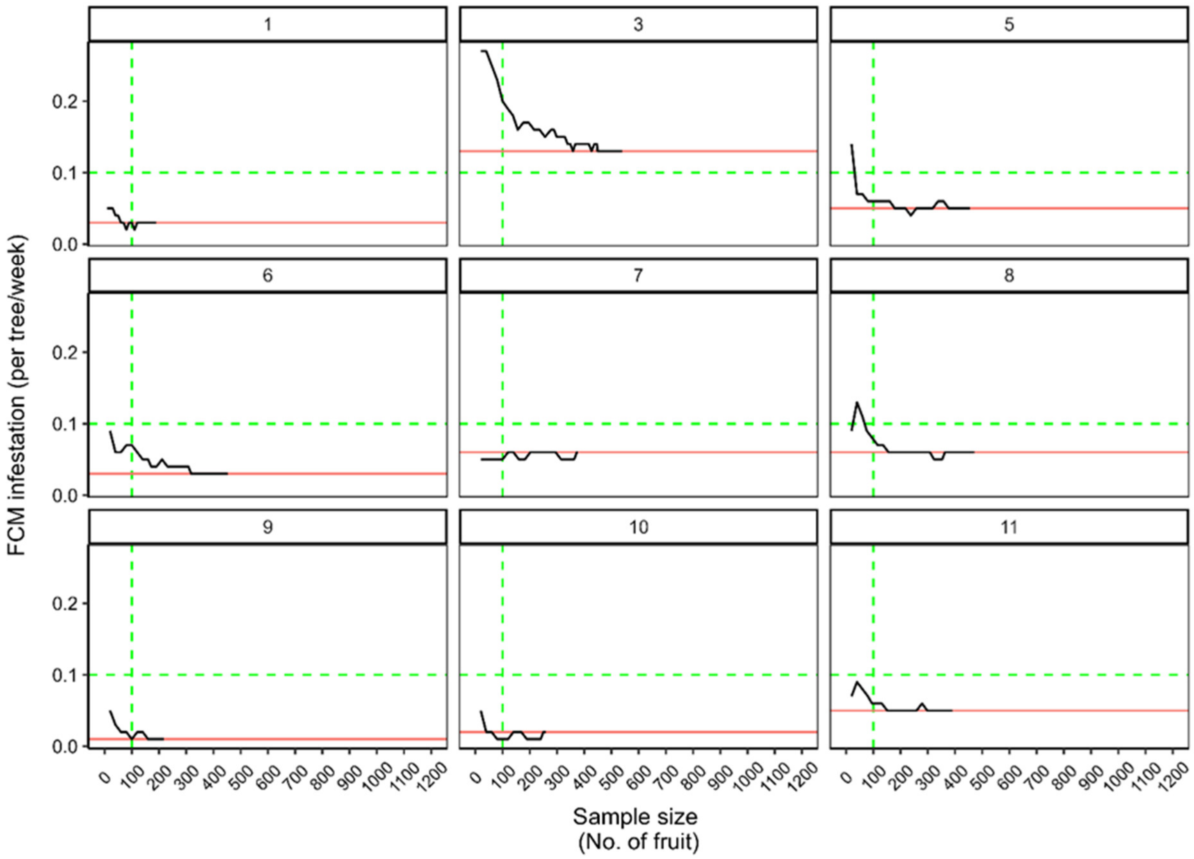

(e) Orchard 21B

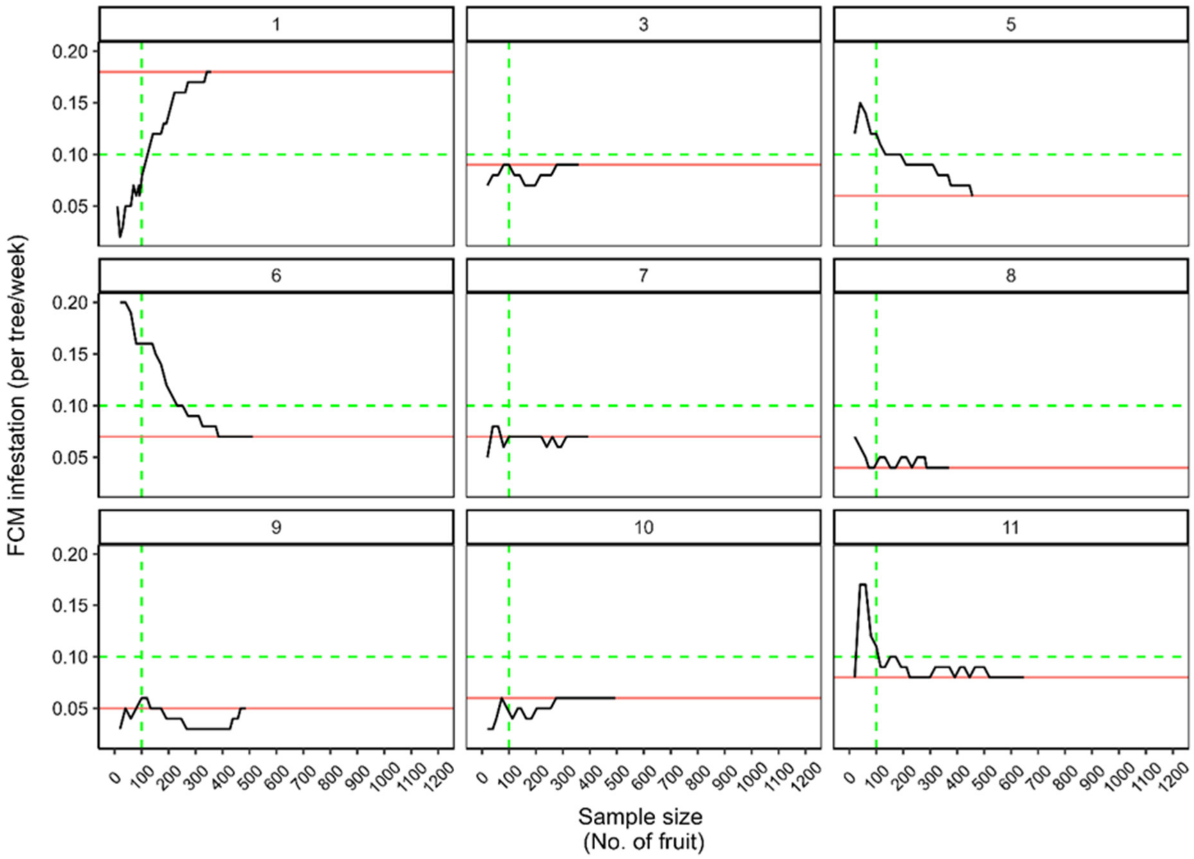

(f) Orchard 22B

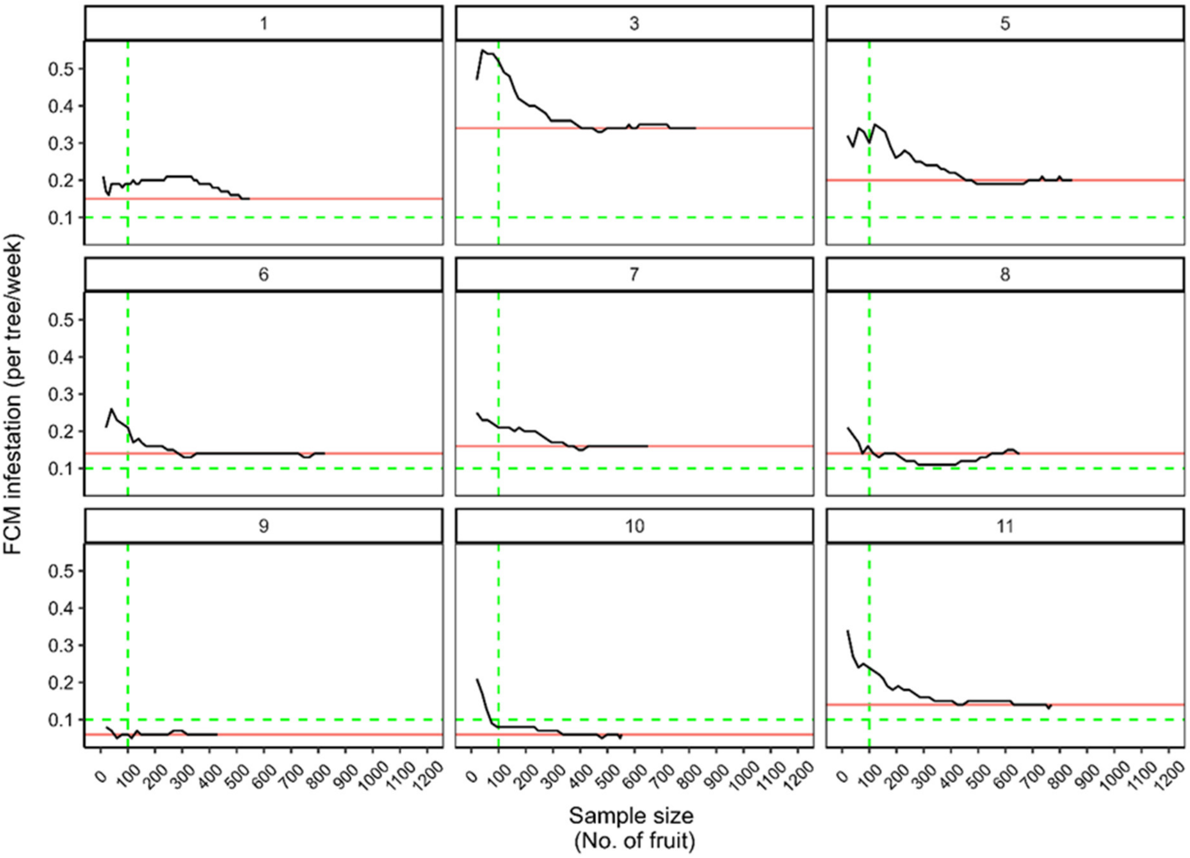

(g) Orchard 23A

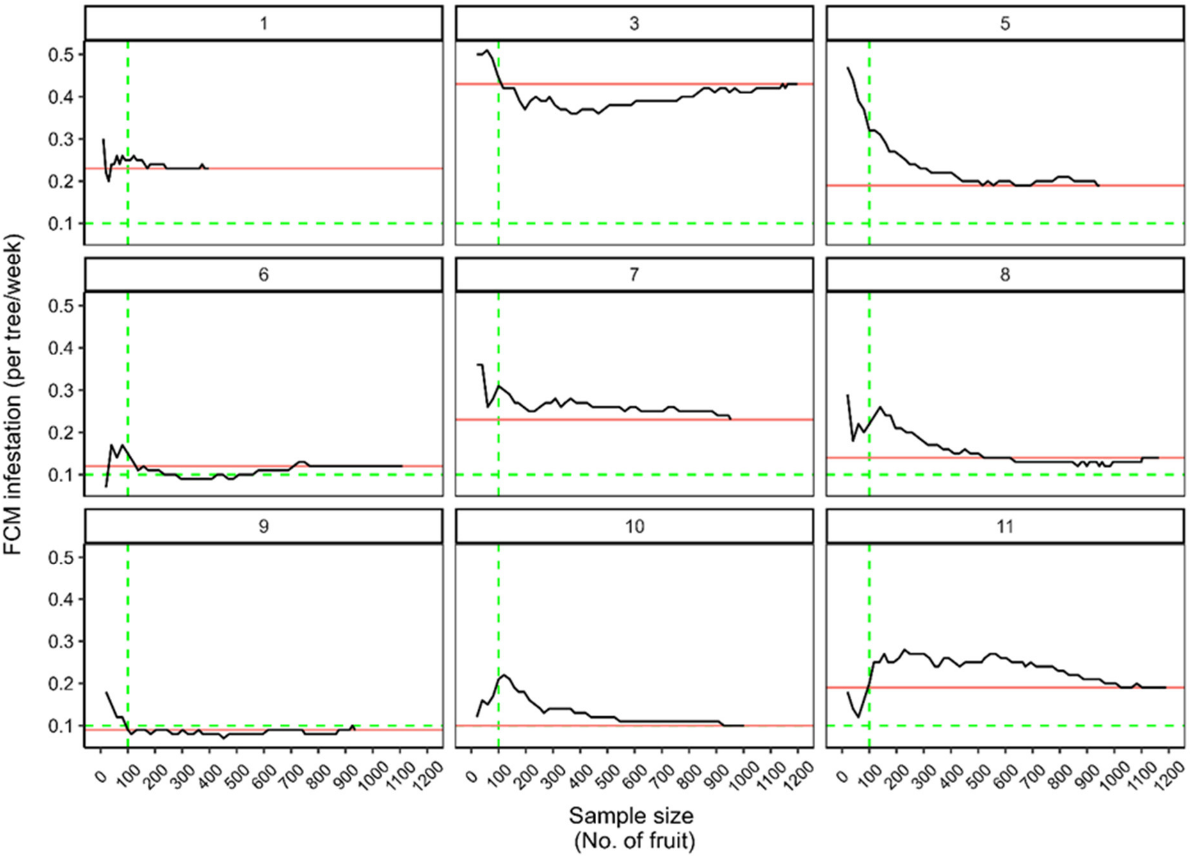

Figure S1 (a – g). Sample size assessment required to provide a reliable measurement of *T. leucotreta* infestation in the sanitation fruit population from the whole orchard for orchards 1 (a), 2 (b), 3 (c), 4 (d), 5 (e), 6 (f), and 7 (g) in 2021. The red line indicates the *T. leucotreta* infestation level in the sanitation fruit population from the whole orchard. The black line indicates *T. leucotreta* infestation level in the subsample of sanitation fruit from the orchard. The vertical green line indicates the first 100 fruit sample, and the horizontal green line indicates the threshold of 0.1 infested fruit per tree per week. The *T. leucotreta* estimate provided by the subsample provided a reliable measurement of the whole orchard infestation level wherever the black line is equivalent to or higher than the red line. The panels indicate different weeks (weeks 1-11, excluding weeks 2 and 4).
